# Prevalence of depression and anxiety symptoms among first-generation medical students

**DOI:** 10.1192/j.eurpsy.2025.924

**Published:** 2025-08-26

**Authors:** S. Aljhani, A. Alhasan, W. Almohaimeed, R. Almeshal, E. Almeshal, I. AlWahhabi

**Affiliations:** 1 Psychiatry; 2 Qassim University, Buraydah, Saudi Arabia

## Abstract

**Introduction:**

Medical students face high demands in college, which may cause significant psychological stress and mental health problems, such as depression and anxiety. Several studies worldwide have shown that such individuals are more likely to experience anxiety. However, few studies have examined how generational status and being a first-generation medical student lead to mental health issues.

**Objectives:**

In this study, we aimed to estimate the prevalence of depression and anxiety in first-generation medical students (FGMS) compared with non-FGMS and to determine the correlation between socioeconomic factors and other variables with depression and anxiety in FGMS.

**Methods:**

This cross-sectional study was conducted among medical students at the College of Medicine. A self-administered questionnaire was distributed to the students using convenience sampling. The questionnaire comprised socio-demographic information (e.g. age, gender, marital status), a General Anxiety Disorder (GAD-7) scale to assess anxiety, and a Patient Health Questionnaire (PHQ-9) to assess depression among medical students.

**Results:**

Among the 309 medical students who completed the questionnaire, 65.4% were female and 75.7% were FGMS. The prevalence of anxiety and depression among medical students was 36.2% and 39.5%, respectively, and was higher among FGMS, but not significantly different (p<0.05). Independent risk factors for anxiety and depression among FGMS included a previous history of mental disorders and lack of social and emotional support, while fair sleep quality was identified as a significant independent preventive factor for anxiety and depression. The prevalence rates of anxiety and depression among patients with FGMS were 39.3% and 41.9%, respectively. A previous diagnosis of mental disorder was a significant risk factor for anxiety and depression, whereas fair sleep quality was a significant protective factor. Further research is needed to identify the factors that influence anxiety and depression among FGMS in our region.

**Conclusions:**

Anxiety and depression are common among first-generation medical students. FGMS with a history of mental disorders tended to exhibit symptoms of both anxiety and depression compared to the rest of the FGMS. However, satisfactory sleep quality could result in better mental condition in FGMS. Institutional measures should be adopted to help students improve their living conditions. Furthermore, institutional leaders should spearhead the destigmatisation of psychological disorders and advocate help-seeking behaviours when students need mental help, particularly when they are anxious or depressed.

**Disclosure of Interest:**

None Declared

